# Quantum Zeno Effect assisted Spectroscopy of a single trapped Ion

**DOI:** 10.1038/s41598-018-28824-w

**Published:** 2018-07-13

**Authors:** Akira Ozawa, Josue Davila-Rodriguez, Theodor W. Hänsch, Thomas Udem

**Affiliations:** Max-Planck-Institute of Quantum Optics, Hans-Kopfermann-Str. 1, D-85741 Garching, Germany

## Abstract

The quantum Zeno effect (QZE) is not only interesting as a manifestation of the counterintuitive behavior of quantum mechanics, but may also have practical applications. When a spectroscopy laser is applied to target atoms or ions prepared in an initial state, the Rabi flopping of an auxiliary transition sharing one common level can be inhibited. This effect is found to be strongly dependent on the detuning of the spectroscopy laser and offers a sensitive spectroscopy signal which allows for high precision spectroscopy of transitions with a small excitation rate. We demonstrate this method with direct frequency comb spectroscopy using the minute power of a single mode to drive a dipole allowed transition in a single trapped ion. Resolving the individual modes of the frequency comb demonstrates that the simple instantaneous quantum collapse description of the QZE can not be applied here, as these modes need several pulses to build up.

## Introduction

In conventional quantum mechanics the dynamics of an unobserved system is described by the time-dependent Schrödinger equation while the dynamics of the observed system is governed by the proclaimed collapse of the wave function. The latter resets all coherences and assigns probabilities to eigenstates of the measurement basis and is best described by a density matrix formalism. Alternatively to the collapsing wave function, one may use a more complete description, by including the measurement procedure into the Schrödinger equation. The “collapse” then becomes part of the system dynamics^1–3^. The loss of coherence is fundamentally due to tracing over unobserved degrees, which are the modes of the spontaneously emitted photons here. A closed system never loses its coherence.

The collapse description can be a good approximation, for example to model the quantum Zeno effect (QZE)^2,4,5^. The condition is that it occurs on a time-scale which is fast compared to the Schrödinger dynamics of the system and that the system shows a quadratic evolution of its occupation probability. In this picture a series of frequent projection measurements continues to collapse the wave function back to the initial state and inhibits the Schrödinger dynamics. For exponential time evolution, which is linear for short times, the Zeno effect does not take place. However, the original proposed inhibition of the decay of an unstable particle due to the Quantum Zeno *paradox* is of that type^6^. In fact spontaneous decay shows a quadratic time-evolution for a very short time on the order of the coherence time of the vacuum fields^7^. So far this has been too short to be observable except in analog systems like tunneling atoms in an accelerated optical lattice^8^. An infinitely frequent observation is required to completely halt any dynamics. This, however, was shown to be unphysical as it would violate the uncertainty principle^9^. Although quantum mechanics gives a statistical description of an ensemble of systems only, it is demonstrated that an individual quantum system also exhibits the Zeno effect^10^. The QZE is also observed in systems with non-exponential dynamics, such as Rabi flopping^4,11^, which is employed in this work.

## Zeno Spectroscopy

**Figure 1:**
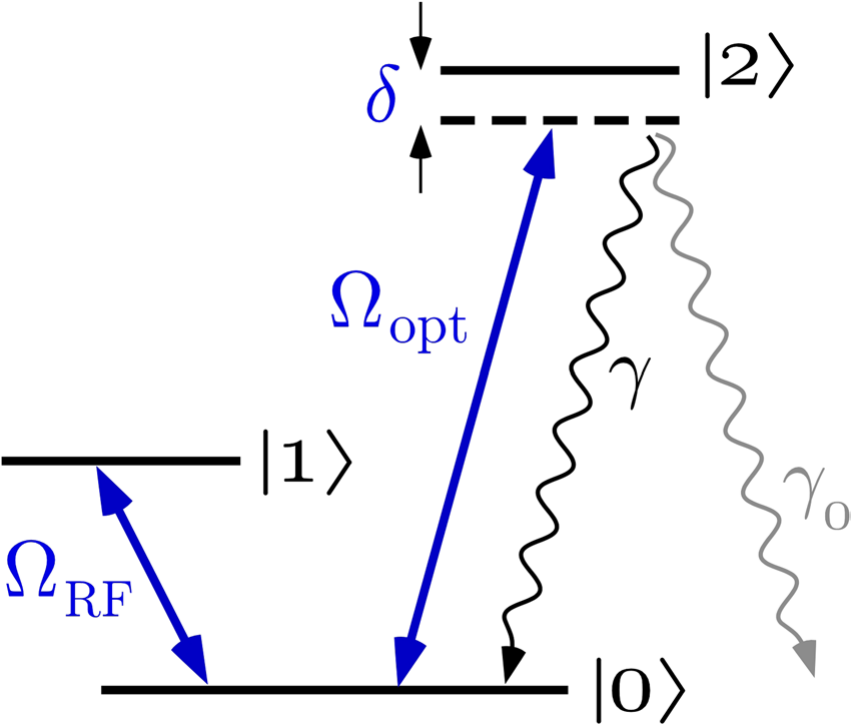
Level scheme used in this work. On resonance Rabi flopping on an auxiliary radio frequency (RF) transition between two ground state hyperfine levels |0〉 and |1〉 is induced with a Rabi frequency Ω_RF_. At the same time the optical transition under investigation |0〉 → |2〉 (or spectroscopy transition) is laser excited with a Rabi frequency Ω_opt_. In contrast to the radio frequency transition, the optical transition is assumed to be damped with decay constants *γ* and *γ*_0_ (possible decay to other states). This decay also leads to a decoherence of the radio frequency transition that depends on the laser detuning *δ* in form of a Lorentzian line shape. The resulting damping of the Rabi flopping Ω_RF_ is used as the signal to detect the |0〉 → |2〉 transition.

In addition to its fundamental interest, the QZE finds practical applications, for example, in improving quantum information processing^12–14^. Zeno spectroscopy, as proposed and demonstrated in a rudimentary way in this work, adds another application with a high detection sensitivity such as electron shelving^15,16^ and quantum logic spectroscopy^17^. A possible level scheme is laid out in Fig. 1. In the unobserved case undamped Rabi flopping at frequency Ω_RF_ between two stable levels |0〉 and |1〉 takes place. A second transition “measures” the occupation of |0〉 by driving a damped transition from |0〉 to |2〉. The presence of the corresponding Rabi frequency Ω_opt_, reveals itself not only by the emitted photons but also by inhibiting or damping the Rabi flopping between the stable ground states. This loss of coherence that the QZE imposed on the |0〉 → |1〉 is used as a signal for performing spectroscopy on the |0〉 → |2〉 transition. If the system is initially prepared in the |0〉 state, the generation of a spontaneous photon projects it back to the original state (QZE). Alternatively, by preparing |1〉 as the initial state, the absence of this photon projects the system back (null-measurement QZE). Note that neither the existence or absence of the photon has to be detected. Also, the decay of the excited state might take place to yet another level.

Our level scheme is very similar to the one used in the famous electron shelving method^15,16^, where the strong fluorescence of a cooling transition is interrupted by a transition to the upper state of a narrow optical transition. The relative orientation of these levels (Vee-, lambda- or ladder) does not matter. Neither does the transition frequencies (optical or RF). The shelving method is applicable when a target transition shares the ground or excited state with a auxiliary transition with broader natural linewidth. Our intended application of the Zeno spectroscopy is the opposite case where the auxiliary transition is narrower than the spectroscopy transition. Even with broader spectroscopy transition, sometimes strong excitation is not possible because of limited laser power. This is especially the case when the spectroscopy transition lies at exotic wavelengths such as the extreme ultraviolet. If properly shielded the RF transition never decoheres unless it is “observed” by the spectroscopy transition. In contrast to the shelving method, we assume that no photons can be detected on the RF transition, but there is another means to detect this transition.

There are several ways for state detection without fluorescence, for example by state dependent loss from an ion trap through an optically pumped beam of Cs atoms. This has been demonstrated a long time ago to prepare and detect the ground state hyperfine state of trapped ^3^He ions^18^. In combination with the thus far very limited power of existing frequency combs at a wavelength of 60.8 nm based on high harmonic generation^19^, this could allow the detection of the extremely narrow 1S–2S transition in ^3^He^+^ for bound state QED tests^20^. No continuous wave cooling laser for shelving with the 30 nm 1S–2P transition seems to be in reach. Another option for photon-less state detection would be to employ a state dependent optical dipole force^21^. We have not yet employed such a technique in this work but use common optical fluorescence to detect the state |0〉 instead as a proof of principle. This is similar to the work of Streed *et al*.^22^ who have investigated a similar setting using a Bose-Einstein condensate, however without discussing the detuning dependence and the possibility for sensitive spectroscopy.

With the simple collapse picture we can not describe the dependence of the signal on the laser detuning *δ* nor a partial loss of coherence^23^, say for large detuning *δ* and/or weak laser intensity that is quantified by Ω_opt_. Since this is essential for using this effect for spectroscopy, a more complete description is required. We use the three level OBEs in the rotating wave approximation^1–3^ for the density matrix *ρ* for this purpose:

1 $${\dot{\rho }}_{00}=-\,{{\rm{\Omega }}}_{{\rm{RF}}}\,{\rm{Im}}({\rho }_{01})-{{\rm{\Omega }}}_{{\rm{opt}}}\,{\rm{Im}}({\rho }_{02})+\gamma {\rho }_{22}$$

2 $${\dot{\rho }}_{11}={{\rm{\Omega }}}_{{\rm{RF}}}\,{\rm{Im}}({\rho }_{01})$$

3 $${\dot{\rho }}_{22}={{\rm{\Omega }}}_{{\rm{opt}}}\,{\rm{Im}}({\rho }_{02})-\gamma {\rho }_{22}$$

4 $${\dot{\rho }}_{01}=i\frac{{{\rm{\Omega }}}_{{\rm{RF}}}}{2}({\rho }_{00}-{\rho }_{11})-i\frac{{{\rm{\Omega }}}_{{\rm{opt}}}}{2}{\rho }_{12}^{\ast }$$

5 $${\dot{\rho }}_{12}=-\,i\delta {\rho }_{12}+i\frac{{{\rm{\Omega }}}_{{\rm{opt}}}}{2}{\rho }_{01}^{\ast }-i\frac{{{\rm{\Omega }}}_{{\rm{RF}}}}{2}{\rho }_{02}-\frac{\gamma }{2}{\rho }_{12}$$

6 $${\dot{\rho }}_{02}=-\,i\delta {\rho }_{02}+i\frac{{{\rm{\Omega }}}_{{\rm{opt}}}}{2}({\rho }_{00}-{\rho }_{22})-i\frac{{{\rm{\Omega }}}_{{\rm{RF}}}}{2}{\rho }_{12}-\frac{\gamma }{2}{\rho }_{02}$$

**Figure 2:**
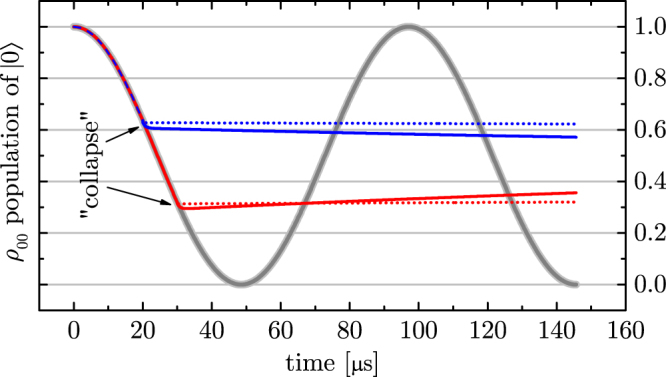
Solving the optical Bloch eqs (1–6) with Rabi flopping at Ω_RF_ = 2*π* × 10.3 kHz and an initial state |0〉. Unobserved dynamics with Ω_opt_ = 0 (grey, thick). At 20 *μ*s (blue, solid) and 30 *μ*s (red, solid) the observing optical transition is added with $\gamma =2\pi \times 41.8\,{\rm{MHz}}\gg {{\rm{\Omega }}}_{{\rm{opt}}}=2\pi \times 4.18\,{\rm{MHz}}\gg {{\rm{\Omega }}}_{{\rm{RF}}}$. According to the “collapse” description this freezes the dynamics which is seen to be a good approximation. It is an even better one for larger *γ* and Ω_opt_. The dotted lines are computed for ten times larger Ω_opt_ and *γ*. Note that the coherences (not shown) are set to zero by the strong optical transition, such that the system is found in either its states with a probably given by the diagonal elements of *ρ*, in accordance with the QZE. The parameters used in this plot do not agree well with the intended application but are instead chosen to match with the rudimentary experimental demonstration presented below.

Here *δ* stands for angular frequency detuning of the spectroscopy laser. These equations may be derived from the more fundamental master equation^7^ by tracing out the modes of the spontaneous photons leading to the otherwise phenomenological damping constant *γ*. Using these equations avoids the simple collapse picture, albeit a very similar dynamics is obtained if the laser measures the state |0〉 quickly and with high fidelity. This is the case for $\gamma \gg {{\rm{\Omega }}}_{{\rm{opt}}}\gg {{\rm{\Omega }}}_{{\rm{RF}}}$ and is shown in Fig. 2 ^24^. We refer to this as the quantum Zeno effect in the sense that observation zeroes the coherences and inhibits dynamics.

**Figure 3:**
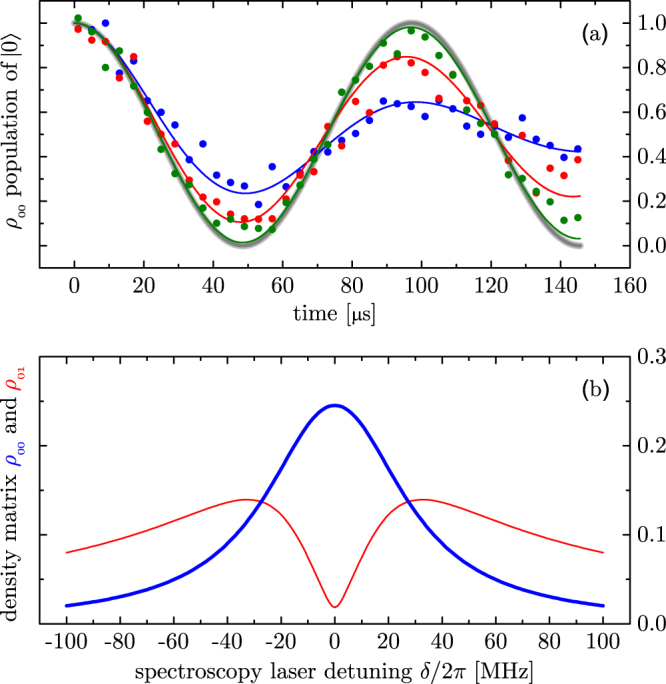
(**a**) Rabi flopping with Ω_RF_ = 2*π* × 10.3 kHz in the unobserved case as in Fig. 2 without the spectroscopy laser on Ω_opt_ = 0 (grey, thick). With a relatively weak spectroscopy laser Ω_opt_ = 2*π* × 0.6 MHz on, the Rabi flopping is not stopped instantaneously as in Fig. 2 but damped out with a time constant that depends on the spectroscopy laser detuning *δ*. Examples are for *δ* = 2*π* × 6.6 MHz, 2*π* × 35 MHz and 2*π* × 122 MHz (blue, red and green respectively). The closer the spectroscopy laser is tuned to resonance, the larger the rate of spontaneous photons that destroy the coherence *ρ*_01_. Parameters, including *γ* = 2*π* × 41.8 MHz, are adapted to be compared with our experimental data (solid circles). (**b**) A Lorentzian line shape is obtained by measuring the occupation *ρ*_00_ of the |0〉 state (blue, thick) after the Rabi flopping between |0〉 and |1〉 has been evolved for a fixed time. In this example this time interval was chosen to correspond to a *π*-pulse. Other choices are possible and may lead to a signal inversion (dip rather than a peak). The magnitude of the detuning dependent coherence *ρ*_01_ is shown in the red (thin) curve.

When detecting a weak transition the condition ${{\rm{\Omega }}}_{{\rm{opt}}}\gg {{\rm{\Omega }}}_{{\rm{RF}}}$ of Fig. 2 is not necessarily given. In this case the “collapse” will take a finite time, possibly several periods of the Rabi flopping between the ground states |0〉 and |1〉. The resulting behavior corresponds to an incomplete “collapse”^23^ or damping of the Rabi flopping (see Fig. 3a). The dependence of this damping on the detuning of the spectroscopy laser *δ* is the basis of Zeno spectroscopy. The signal is constructed by preparing either the |0〉 or the |1〉 state and then driving both transitions for certain time interval. Subsequently, the detuning dependent occupation *ρ*_00_ (or *ρ*_11_) is recorded. Neither the detuning dependence nor the partial “collapse” can be easily modelled by the quantum projections at random times as in ref.^4^. To get a rough idea what is happening, we combine eqs (2 and 4) to obtain ${\ddot{\rho }}_{11}={{\rm{\Omega }}}_{{\rm{RF}}}^{2}({\rho }_{00}-{\rho }_{11})\mathrm{/2}-{{\rm{\Omega }}}_{{\rm{RF}}}{{\rm{\Omega }}}_{{\rm{opt}}}\,{\rm{Re}}({\rho }_{12})\mathrm{/2}$. Without the optical field (Ω_opt_ = 0) this leads to usual undamped Rabi flopping between the states |0〉 and |1〉. Turning on the optical field leads to a damping via the *ρ*_12_-term that is detuning dependent. This is the signal that is recorded. An approximate expression for *ρ*_12_ for the limit ${{\rm{\Omega }}}_{{\rm{opt}}}\ll \gamma ,{{\rm{\Omega }}}_{{\rm{RF}}}$ is given in the appendix and leads to a Lorentzian line shape as seen in Fig. 3b. At the line center the coherence *ρ*_01_ is lost almost completely (under the condition of Fig. 3). For this to happen, the spectroscopy laser and the transition can indeed be very weak. It needs to provide only one scattered photon per half Rabi cycle and hence has the same sensitivity as electron shelving (see appendix). This is consistent with the wavefunction collapse picture that coherence is lost after scattering a single photon. The decoherence-assisted spectroscopy that was performed by Clos it *et al*.^25^ is yet another spectroscopy method based on the decoherence. The decoherence-assisted spectroscopy detects the loss of the coherence in the auxiliary levels that were initially prepared to a certain coherent superposition. The Zeno spectroscopy is based on a similar mechanism, but utilizes the suppression of any coherent dynamics in the auxiliary levels. Therefore it can be considered as a generalization and extension of the decoherence assisted spectroscopy.

To be able to use the Zeno spectroscopy for high precision metrology, it is important to understand possible line shape distortions beyond the approximations made here. Investigating the OBEs one finds that flipping the sign of the detuning *δ* can be undone by replacing ${\rho }_{01}\to -\,{\rho }_{01}^{\ast }$, ${\rho }_{02}\to -\,{\rho }_{02}^{\ast }$ and ${\rho }_{12}\to {\rho }_{12}^{\ast }$. This means that the dynamics of the coherences is modified, but the diagonal elements of the density matrix are not. Hence the line shape is perfectly symmetric for any choice of parameters such that no spurious line shifts are expected when fitted with any other symmetric line shape. Of course this leaves aside possible asymmetric distortions and shifts due to additional levels not included in our OBEs that give rise to the ac-Stark shift for example.

Line broadenings are observed due to commonly known mechanisms. When the probe time *τ* gets shorter or comparable to the life time of the excited state, the usual probe time broadening takes place. As long as ${{\rm{\Omega }}}_{{\rm{RF}}}\tau \ll 1$, power broadening just like in a two level system occurs (see appendix). Failing to fulfill this condition results in a line width that is significantly larger than simple power broadening with a more complex (but symmetric) structure. All of this follows from numerical solutions of the OBEs (1–6). At high intensities or long probe times decoherence may take place even for very large detuning, which results in a very broad spectroscopy signal. This feature might be used for an initial search of a narrow line of uncertain frequency such as the ^229^Th optical nuclear transition^26^.

## Apparatus

**Figure 4:**
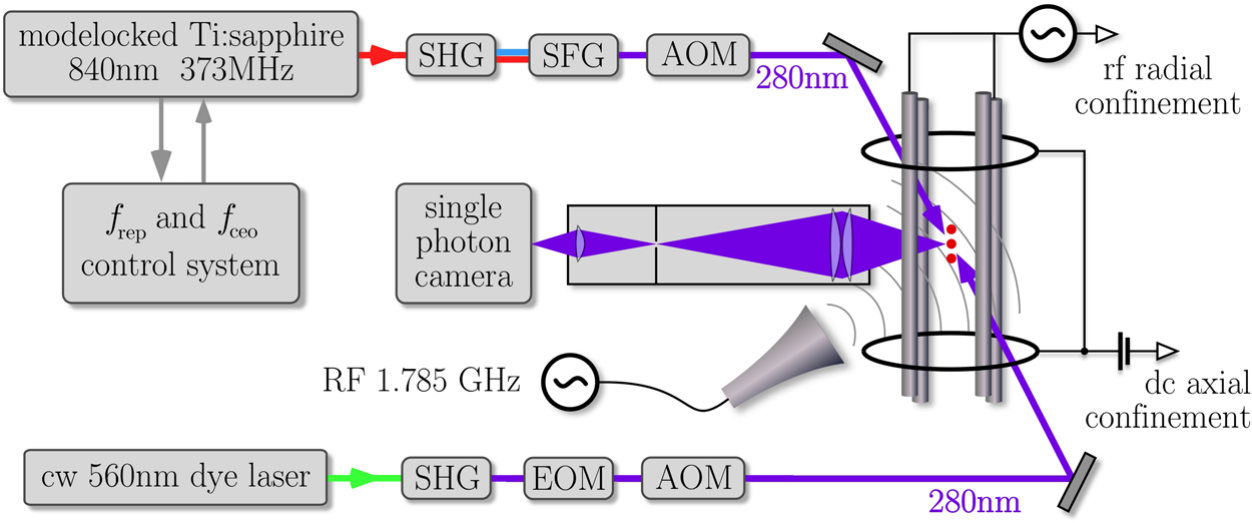
Schematic of the experimental setup. SHG: second-harmonic generation stage, SFG: sum-frequency generation stage, EOM: electro-optic modulator, AOM: acousto-optic modulator.

Ions confined and isolated in a Paul trap are ideal targets to investigate the Zeno-dynamics because they experience little disturbances from the environment and electric/magnetic field^4,27^. To demonstrate the Zeno spectroscopy in a rudimentary way we use our previously described ion trap that is equipped with a deep-ultraviolet frequency comb at around 280 nm and a continuous wave (cw) cooling/preparation laser at the same wavelength^28^. In a real application one would rely on sympathetic cooling instead. Schematic of the experimental setup is shown in Fig. 4. The trap is a four-rod Paul trap operating at 22 MHz with a radial secular frequency of ~1 MHz. Ions are loaded into the trap by evaporating neutral magnesium and photo-ionizing them via resonant excitation with a pulsed dye laser at 570 nm, which is frequency doubled to 285 nm. By tuning the photoionization laser we can make the process isotope selective. The selection is found to be imperfect because of the large linewidth of our photo-ionization laser and the Doppler broadened profile of the Mg vapor. We typically load 1…3 ions and repeat the loading process until a pure sample of ^25^Mg^+^ is obtained. It is found that repeatedly flashing the trap voltage introduces heating of the trapped ions that removes dark ions, mostly ^24,26^Mg^+^ and magnesium hydride ions, more efficiently than the laser cooled ^25^Mg^+^. The odd isotope has a nuclear spin of *I* = 5/2 and such that the 3*s*_1/2_ ground state splits into *F* = 2 and *F* = 3 angular momentum states.

Three pairs of Helmholtz-coils are installed around the ion trap to cancel the stray magnetic field. The same coils are used to apply a small magnetic field (~1.8 Gauss) along the cooling and spectroscopy beams to define the quantization axis. The spectroscopy laser is a frequency comb at 280 nm generated by frequency tripling a Ti:sapphire mode-locked ring laser that has a repetition rate of 373 MHz. Both the repetition and offset frequencies are locked to local oscillators which are phase-locked to a GPS-disciplined hydrogen-maser, ensuring traceability of the frequency of each comb-line^29^. The frequency comb and cw laser are switched by acousto-optic modulators (AOMs) with rise/fall switching-time of ~1 *μ*s. An electro-optic modulator (EOM) is used to generate a sideband for repumping (see below). The AOMs and EOM are controlled by real-time I/O system (ADwin Pro-II) to obtain desired pulse sequences.

Fluorescence from the ions is collected by a *f*/2 four-lens condenser at a window close to the trap center and then imaged on the sensor plane of a micro channel plate single photon camera (Quantar Technologies, Mepsicron II) with a microscope objective. The camera has sub-ns time resolution that allows us to record the fluorescence events along with their arrival times which, in turn, permits us to distinguish the state preparation and state detection periods. By selecting the regions of interest from the camera images we can evaluate the data from the ions individually and in parallel. In determining the centroid frequency, the magnetic-field component along the spectroscopy beam is measured by performing radio frequency spectroscopy of the ground state magnetic sublevels. The mode number of the comb is determined using our previous spectroscopy results^30,31^.

## Experimental Demonstration

We choose the 3*s*_1/2_(*F* = 3, *M* = 3) and the 3*s*_1/2_(*F* = 2, *M* = 2) as the two ground states |0〉 and |1〉 respectively. The |0〉 state can be readily prepared via optical pumping by exciting the D_2_ line (cycling transition of 3*s*_1/2_(*F* = 3, *M* = 3) → 3*p*_3/2_(*F* = 4, *M* = 4) in combination with a repumper sideband to prevent population trapping in the 3*s*_1/2_(*F* = 2) levels.

**Figure 5:**
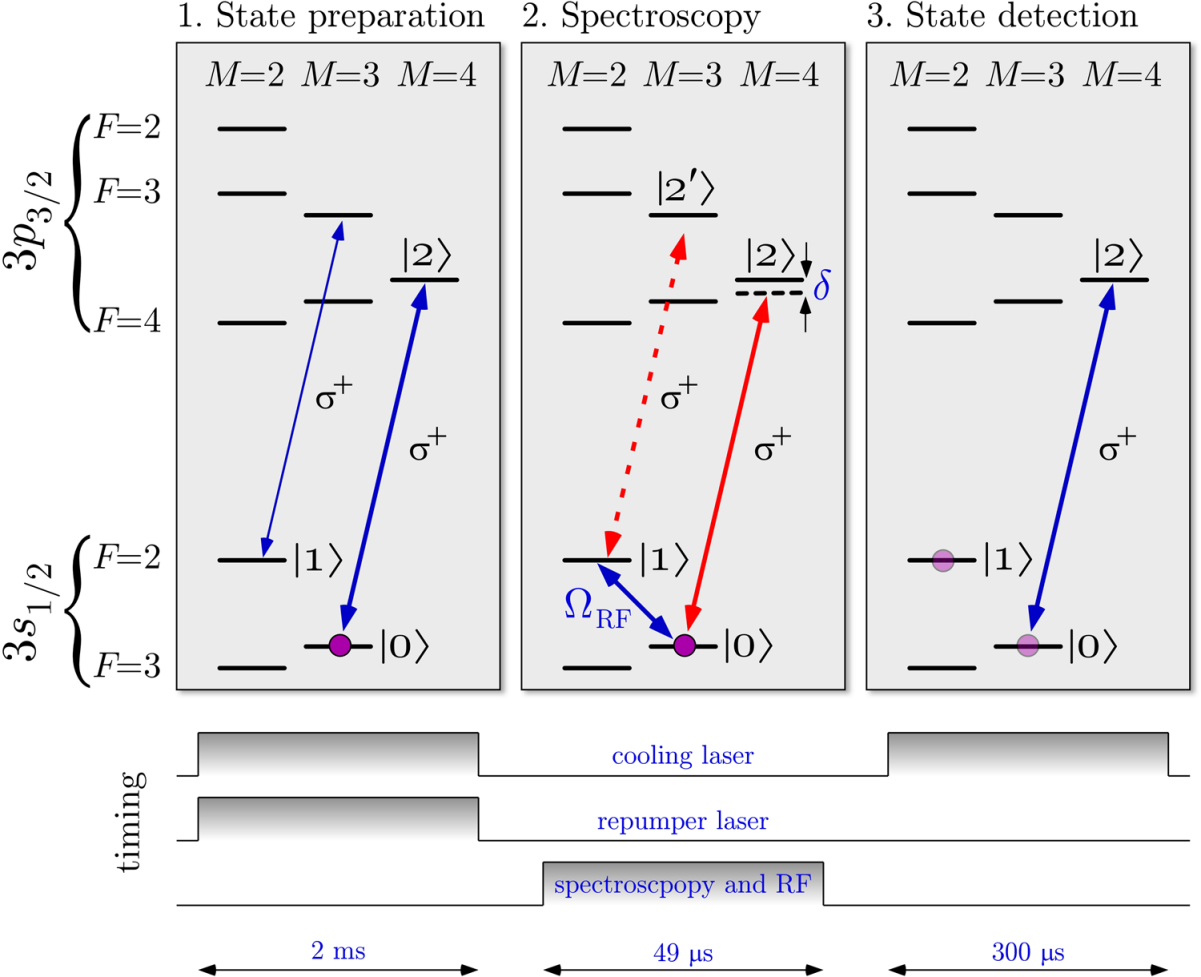
In the first step the ion is prepared (and cooled) in the |0〉 = 3*s*_1/2_(*F* = 3, *M* = 3) state by optical pumping with a continuous wave laser. Then Zeno spectroscopy is performed by observing the damping of Rabi flopping of the |0〉 = 3*s*_1/2_(*F* = 3, *M* = 3) → |1〉 = 3*s*_1/2_(*F* = 2, *M* = 2) driven with a radio frequency of 1.785 GHz (see Fig. 3). Zeno spectroscopy also works for the non-cycling transition to |2′〉 (dashed). Since the spectroscopy laser in this case is a frequency comb, we see both transitions convolved with the comb (see Fig. 6). In the last step the population remaining in the initial state is determined. At this point we simply use the same laser as cooling/preparation laser on its cycling transition for a demonstration whereas in a real application such a laser would not exist (otherwise the usual shelving or fluorescence detection scheme seem to be simpler). The preparation and state detection would have to be done by other means such as state dependent trap losses^18^.

Each experiment consists of a sequence as shown at the bottom of Fig. 5, where there is a long (~2 ms) cooling period with both the cooling and repumper lasers shining on the ions and then a spectroscopy period is present with a *π*-pulse of the radio frequency transition. Data recorded with a variable spectroscopy period is shown in Fig. 3 for a few representative detunings. We observe a spurious decay of coherence with a time constant of 1.2 ms presumably due to stray magnetic fields. This generates an additional constant background of our line shapes. In the state detection period, the population of |0〉 is measured via the cycling transition of the ^25^Mg^+^ D_2_ line. Collected fluorescence counts are normalized to that of the unperturbed ion detected during the state preparation period. We choose to use a frequency comb as a spectroscopy laser because it can readily demonstrate the main advantages of the technique, which are higher sensitivity and smaller background noise compared with direct fluorescence detection scheme.

The optimum amount of comb power for spectroscopy in order to obtain well-resolved narrow lines was much lower than the power available. Under our experimental conditions, we found that even ~50 *μ*W of average power of frequency comb, which corresponds to approximately 1.5 nw per comb mode, was sufficient to introduce a significant QZE. Larger comb power can be used to broaden the lines and optimizing the alignment of the cooling laser and the comb. The observation is in contrast with direct fluorescence collection method for which the entire comb power and longer measurement time had to be employed to obtain a comparable signal-to-noise ratio^31^.

**Figure 6:**
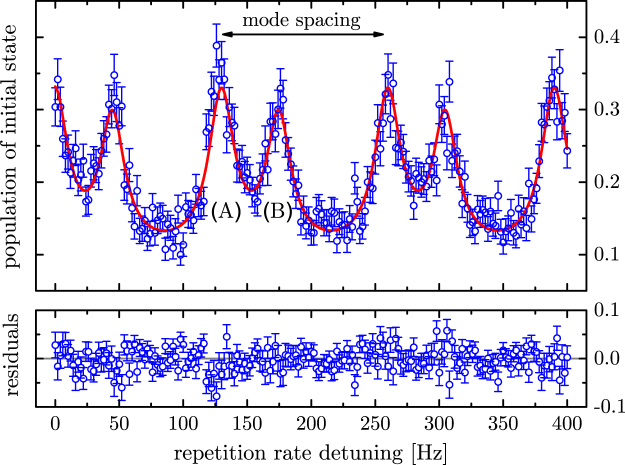
Typical spectroscopy signal obtained by Zeno spectroscopy with a frequency comb. The remaining population of the initial state after the spectroscopy period is plotted for different detunings, which is a measure of the strength of the QZE. The horizontal axis shows repetition rate detuning from 373, 258, 500 Hz. The repeating structure has a period that corresponds to the mode structure of the frequency comb. The red line shown is a fit with two Lorentzian curves (two transitions shown in Fig. 5) convolved with the comb spectrum. Poissonian noise is assumed for the fluorescence signal. Peaks at (A) and (B) correspond to 3*s*_1/2_(*F* = 3, *M* = 3) → 3*p*_3/2_(*F* = 4, *M* = 4) and 3*s*_1/2_(*F* = 2, *M* = 2) → 3*p*_3/2_(*F* = 3, *M* = 3) transitions respectively.

A scan over several comb-lines of the spectroscopy is shown in Fig. 6. The spectrum is periodic with the mode spacing of the comb. Pairs of lines are observed which correspond to 3*s*_1/2_(*F* = 3, *M* = 3) → 3*p*_3/2_(*F* = 4, *M* = 4) and 3*s*_1/2_(*F* = 2, *M* = 2) → 3*p*_3/2_(*F* = 3, *M* = 3) (see Fig. 5). The latter transition corresponds to the null-measurement case. In this case it is possible to perform spectroscopy without exciting the target transition, similarly to interaction free measurement^32^.

To evaluate our data we fitted two Lorentzians convolved with the comb spectrum in accordance with the approximations made in the appendix. The more rigorous treatment with the OBEs (1–6) gives very little deviation from the Lorentzian model. Assuming a Poisson distribution for the signal counts, the statistical uncertainty of the line center would be 1 MHz. However, in our particular implementation of the Zeno spectroscopy there are more levels addressed than accounted for by (1–6). Expanding to the four-level OBEs by adding the |2′〉 level of Fig. 5 indeed leads to a line shift that is smaller than 1 MHz.

Another systematic effect is due to the spectral tail of the comb that also addresses the D_1_ line. The 3*s*_1/2_(*F* = 2, *M* = 2) → 3*p*_1/2_(*F* = 3, *M* = 3) transition may contribute to the signal, although it is not clearly visible in the obtained spectra. We model this by generating artificial data sets that includes this component with a variable position and an amplitude given by our detection limit. Fitting the same function as used to evaluate the real data we estimated this shift to be smaller than 6 MHz. Note that many of these effects are artifacts of using frequency combs as an excitation laser and not a fundamental limitation of the Zeno spectroscopy scheme. Considering above systematic effects, the centroid frequency of the 3*s*_1/2_ → 3*p*_3/2_ transition is obtained to be 1 072 084 553(6) MHz. This value is consistent with previous measurements^25,30,31^ and thus shows the applicability of the Zeno spectroscopy for high precision spectroscopy.

## The Collapse Again

The spectrum of frequency combs can be considered as a collection of mutually phase-coherent narrow lines, which explains the spectroscopy signal that repeats itself with mode-spacing as shown in Fig. 6. On the other hand, it should be noted that the frequency comb consists of a periodic train of short pulses in the time domain. If we employ the projection postulate in describing the QZE driven by frequency combs, one would naively consider that each individual pulse performs a quantum measurement. Since the mode structure of frequency comb originates from coherent interference between the pulses, this picture fails in describing our spectroscopy results because the individual comb modes are resolved. Therefore, in our experiment, the “measurement” should be considered to occur over many pulses of the mode-locked laser, which is always the case when the pulse-to-pulse interval is shorter than the upper-state life-time of the target transition. So far, many experimental demonstrations of the QZE could be explained conveniently by both the frequent quantum measurement picture and the decoherence picture using the OBE. Our demonstration is an example where the approximate description with the simple collapse picture fails.

## Appendix

To derive the line shape of the Zeno spectroscopy for the system shown in Fig. 1 we use the optical Bloch equations (OBE) (1–6) in the limit of $\gamma \gg {{\rm{\Omega }}}_{{\rm{opt}}},{{\rm{\Omega }}}_{{\rm{RF}}}$. With the large damping we can assume that *ρ*_22_, *ρ*_02_ and *ρ*_12_ follows the dynamics on the |0〉 → |1〉 transition adiabatically. By setting the left hand side of (3), (5) and (6) to zero and solve the resulting linear equations while neglecting Ω_opt_ and Ω_RF_ against *γ* gives:

7 $${\rho }_{12}^{\ast }=-\,\frac{1}{4}\frac{{{\rm{\Omega }}}_{{\rm{RF}}}{{\rm{\Omega }}}_{{\rm{opt}}}}{{(\delta +i\gamma \mathrm{/2})}^{2}}{\rho }_{00}+\frac{1}{2}\frac{{{\rm{\Omega }}}_{{\rm{opt}}}}{\delta +i\gamma \mathrm{/2}}{\rho }_{01}$$

Using this in (4) gives

8 $${\dot{\rho }}_{01}=-\,i\frac{{{\rm{\Omega }}}_{{\rm{RF}}}}{2}{\rho }_{11}+i\frac{{{\rm{\Omega }}}_{{\rm{RF}}}}{2}(1+\frac{1}{4}\frac{{{\rm{\Omega }}}_{{\rm{opt}}}^{2}}{{(\delta +i\gamma \mathrm{/2})}^{2}}){\rho }_{00}-\frac{i}{4}\frac{{{\rm{\Omega }}}_{{\rm{opt}}}^{2}}{\delta +i\gamma \mathrm{/2}}{\rho }_{01}$$

Neglecting the second term inside the brackets against 1 in accordance to the above limit and assuming that *ρ*_00_ + *ρ*_11_ ≈ 1 due to the fast decay of the |2〉 level and weak spectroscopy laser, we obtain optical Bloch equations that are formally equivalent to the two level optical Bloch equations for the states |0〉 and |1〉:

9 $${\dot{\rho }}_{11}={{\rm{\Omega }}}_{{\rm{RF}}}\,{\rm{Im}}\,{\rho }_{01}$$

10 $${\dot{\rho }}_{01}=-\,i\delta ^{\prime} {\rho }_{01}+i\frac{{{\rm{\Omega }}}_{{\rm{RF}}}}{2}({\rho }_{00}-{\rho }_{11})-\frac{\gamma ^{\prime} }{2}{\rho }_{01}$$

with an effective detuning (actual detuning on the |0〉 → |1〉 is zero) and an effective decoherence rate:

11 $$\delta ^{\prime} =\frac{\delta }{4}\frac{{{\rm{\Omega }}}_{{\rm{opt}}}^{2}}{{\delta }^{2}+{(\gamma \mathrm{/2)}}^{2}}\,{\rm{and}}\,\gamma ^{\prime} =\frac{\gamma }{4}\frac{{{\rm{\Omega }}}_{{\rm{opt}}}^{2}}{{\delta }^{2}+{(\gamma \mathrm{/2)}}^{2}}$$

Note that with the Zeno spectroscopy we are not simply detecting one of the occupations *ρ*_00_ or *ρ*_11_ but the change of the latter due to the presence of the spectroscopy laser. There are analytic solutions of (9, 10) that are simple for *γ*′ = *δ*′ = 0. An analytic solution also exists for the general case, but this is rather lengthy so we are not reproducing it here. For our purpose it is sufficient to expand this solution for short integration times ${{\rm{\Omega }}}_{{\rm{RF}}}\,\tau \ll 1$ with initial conditions *ρ*_00_ = 1:

12 $${\rho }_{11}=\frac{{{\rm{\Omega }}}_{{\rm{RF}}}^{2}}{4}{\tau }^{2}-\gamma ^{\prime} \frac{{{\rm{\Omega }}}_{{\rm{RF}}}^{2}}{24}{\tau }^{3}+\ldots $$

The first term belongs to the unperturbed Rabi oscillations ${\rho }_{11}={\sin }^{2}({{\rm{\Omega }}}_{{\rm{RF}}}\tau \mathrm{/2)}\approx {{\rm{\Omega }}}_{{\rm{RF}}}^{2}\,{\tau }^{2}\mathrm{/4}$ while the second term is due to the Zeno effect. The latter is our signal and seen to be a Lorentzian with a spectral width given by the natural line width of the |0〉 → |2〉 transition.

To estimate the sensitivity of the Zeno spectroscopy we first approximate the steady state population on resonance ${\rho }_{22}\approx {{\rm{\Omega }}}_{{\rm{opt}}}^{2}/{\gamma }^{2}$ by assuming the RF transition does not perturb the |0〉 ↔ |2〉 transition too much. The photon scattering rate on that transition is ${\rho }_{22}\gamma \approx {{\rm{\Omega }}}_{{\rm{opt}}}^{2}/\gamma$ and hence the time required to scatter one photon is given by ${\tau }_{p}={({{\rm{\Omega }}}_{{\rm{opt}}}^{2}/\gamma )}^{-1}$. With *τ* > *τ*_*p*_ the second term in (12) becomes comparable to the first one and the Zeno effect is noticeable. Therefore the Zeno spectroscopy, just like the shelving scheme, possesses the ultimate sensitivity of being able to observe single photon events on the optical transition. In practice though, the sensitivity of both methods depend on the signal to noise ratio in the detection of the auxiliary transition.

### Data availability

The data that support the findings of this study are available from the corresponding author upon reasonable request.
